# Interactions, transformation, and functionality: Lactic acid bacteria fermentation of egg liquid as a novel food matrix

**DOI:** 10.1016/j.fochx.2026.104292

**Published:** 2026-08-08

**Authors:** Yang Zhou, Zhiwen Deng, Siyu Dai, Ruohan Yang, Yutong Xue, Huanhuan Wei

**Affiliations:** a Department of Food Science and Engineering, School of Food and Liquor Engineering, Sichuan University of Science and Engineering, Sichuan, PR China

**Keywords:** Egg liquid, Lactic acid bacteria fermentation, Biotransformation, Structural modification, Physicochemical and functional properties

## Abstract

Eggs are predominantly consumed as basic food ingredients, with limited application in value-added products. Lactic acid bacteria fermentation offers a promising strategy to transform egg matrices into functional food ingredients. This review provides a comprehensive and critical overview of how LAB drives substrate consumption—including proteins, lipids, and carbohydrates—generates bioactive metabolites such as organic acids, flavor compounds, and free amino acids, and induces structural rearrangements in egg proteins at both macroscopic and molecular levels. These changes collectively improve functional properties including solubility, texture, rheology, emulsifying capacity, and foaming stability via mechanisms involving proteolysis, pH-driven aggregation, and redox balance. Importantly, fermentation outcomes are highly condition-dependent—the direction and extent of changes depend on substrate composition, protein concentration, pH, and strain selection. By integrating substrate transformations, structural modifications, and functional outcomes into a unified framework, this review provides mechanistic insights for developing LAB-fermented egg products with enhanced quality and health-promoting potential.

## Highlights

• Provides an integrated map of substrates, LAB, structure, and functional changes.
• Explains condition-dependent effects of LAB on egg protein structure and function.
• Reveals interplay between structural changes and physicochemical properties.

## 1 Introduction

Egg liquid, encompassing egg white (EW), egg yolk (EY), and whole egg (WE), constitutes a foundational ingredient in the food industry (Shi et al., 2024). Its unique physicochemical profile, primarily defined by a complex matrix of proteins and lipids, confers superior gelling, emulsifying, and foaming capabilities, alongside high nutritional value. Concurrently, lactic acid bacteria (LAB) fermentation is a well-established paradigm for modifying texture, flavor, and bioactivity in dairy (Deng et al., 2022).

In contrast, the application of LAB fermentation to egg-derived matrices represents an emerging frontier. While eggs are nutrient-dense, their composition presents distinct challenges for microbial fermentation. Unlike milk, egg liquid is naturally deficient in readily fermentable carbohydrates. Moreover, egg proteins' high molecular weight and complex structures inherently limit LAB growth (Bie et al., 2025; Lv et al., 2024). Despite these constraints, research shows that LAB can survive and actively metabolize in egg-based systems through optimized carbon sources, pH modulation, and matrix dilution (Zang, Pan, et al., 2023). This microbial activity drives protein/lipid hydrolysis, organic acid and flavor compound generation, and structural rearrangements, tailoring egg product functionality (Wang et al., 2022).

Current research on the LAB–egg matrix system has begun to map these transformations, with studies increasingly detailing nutritional components, protein structures, and physicochemical properties (PP). However, four unique technological challenges distinguish egg-based fermentation from dairy systems: (i) insufficient fermentable carbohydrates, requiring exogenous supplementation; (ii) the high molecular weight and complex structures of egg proteins, which are poorly susceptible to LAB proteases and lipases; (iii) naturally occurring antimicrobial components (e.g., lysozyme) that may inhibit LAB growth; and (iv) the narrow window of protein concentration and dilution that determines whether fermentation improves or impairs gel hardness and viscosity.

This review pursues four interconnected objectives: (i) to delineate the fundamental composition and techno-functional attributes of EW and EY; (ii) to outline the established health-promoting and technological functions of LAB in food fermentation contexts; (iii) to critically synthesize current knowledge on how LAB fermentation drives substrate utilization, protein structural rearrangements, and the consequent physicochemical property changes in egg liquid; and (iv) to offer a forward-looking perspective on exploiting the LAB–egg matrix system for innovative product development. The novelty of this review lies in its integration of substrate transformations, structural changes, and functional property alterations into a unified conceptual framework. It also underscores the condition-dependent nature of fermentation outcomes, a perspective not previously articulated. This review connects established findings with identified knowledge gaps to establish a comprehensive framework. This framework guides future research toward exploiting the full potential of LAB-fermented egg products, particularly for developing innovative functional foods and targeted nutrient delivery systems.

## 2 Composition-property relationships of egg liquid

WE comprises two principal components, EW (∼67% *w*/w) and EY (∼33% w/w), with distinct compositions dictating their PP (Khalid et al., 2024; Razi et al., 2023). Both components are strategically used in food applications based on these functions.

### 2.1 Chemical components and PP of EW

EW is predominantly composed of water (88% *w*/w) and protein (11%), with trace lipids, minerals and carbohydrates (<1%) (Razi et al., 2023). The protein fraction of EW consists of ovalbumin (∼54%) (Eastham & Leman, 2024), ovotransferrin (12%), ovomucoid (11%), G2/G3 ovoglobulins (4%), ovomucin (3.5%), and lysozyme (3.4%) (Ji et al., 2020). Lipids account for only 0.02%, while mineral components (K, Ca, Na, Mg, Fe, S, Cl, P) are present in low concentrations. Carbohydrates consist of free glucose (0.4%) and glycoconjugate-bound (0.5%) states (Lee & Paik, 2019).

The distinct composition of EW underpins its techno-functional properties, which are critical in food applications. Its high protein content (∼11% *w*/w) confers superior viscoelasticity and gelling capacity relative to low-protein systems (Bertho et al., 2022). The conformational flexibility of EW proteins allows unfolding upon heating, pH shifts, or shear, facilitating gel network formation (Zang, You, et al., 2023. Zhang et al., 2023). Their interfacial adsorption also enhances foam stabilization (Qing et al., 2024), while their amphipathic nature ensures excellent emulsifying ability (Lafeta et al., 2021).

### 2.2 Chemical components and PP of EY

EY contains water (∼50.2% *w*/w), lipids (∼32% w/w), and proteins (∼16% w/w). The lipid fraction consists mainly of triglycerides (65–70%), phospholipids (25–30%), and cholesterol (4–5%) (Yan et al., 2010). The protein profile is dominated by low-density lipoproteins (LDL, 68%), high-density lipoproteins (HDL, 16%), and phosvitin (14%). These primary components determine nutritional and interfacial characteristics (Yan et al., 2010).

The viscoelastic behavior of EY, arising from its high lipid/lipoprotein content, renders it a key structural modulator in emulsions and foams (Tian et al., 2021; Zhong et al., 2024). Phosphatidylcholine-rich phospholipids act as amphiphilic surfactants at oil-water interfaces. Their hydrophobic tails associate with lipids, while hydrated choline headgroups provide electrostatic repulsion to stabilize emulsions (Bao et al., 2017). Although EY proteins can entrap air via unfolding, lipids generally destabilize foam, giving EY inferior foaming capacity compared with EW (Laca et al., 2014).

## 3 Health-promoting properties of LAB: Background and potential relevance to egg-based fermentation

LAB are widely recognized for multiple probiotic functions including protein digestion, viscosity enhancement, gut microbiota modulation, and immune regulation (Ayed et al., 2024; Dhakal et al., 2024; Zhou et al., 2019). These benefits have been extensively documented in dairy fermentation systems, where LAB metabolize lactose and hydrolyze casein (Deng et al., 2022). However, direct evidence for health-promoting effects of LAB-fermented egg products is currently absent, as most studies have focused on physicochemical changes rather than in vivo activities.

Nevertheless, the metabolic transformations occurring during LAB fermentation of egg matrices produce several bioactive compounds. LAB fermentation of WE generates short-chain fatty acids (SCFAs) such as acetate and butyrate (Gao et al., 2025), as well as free amino acids and low-molecular-weight peptides (∼5 kDa) (Jiang et al., 2020). SCFAs are recognized for their roles in nourishing colonocytes, modulating gut microbiota, and exerting anti-inflammatory effects (Kim et al., 2024). Therefore, LAB-fermented egg products hold promising but yet-to-be-demonstrated bioactive potential, warranting dedicated future investigations.

This section does not claim existing evidence but provides background and metabolic rationale for expecting similar benefits. Future research should directly evaluate the in vivo health-promoting activities of LAB-fermented egg products, including gut microbiota modulation, immune regulation, and digestive health.

## 4 Effects and mechanisms of LAB fermentation on material transformations of egg

**Fig. 1:**
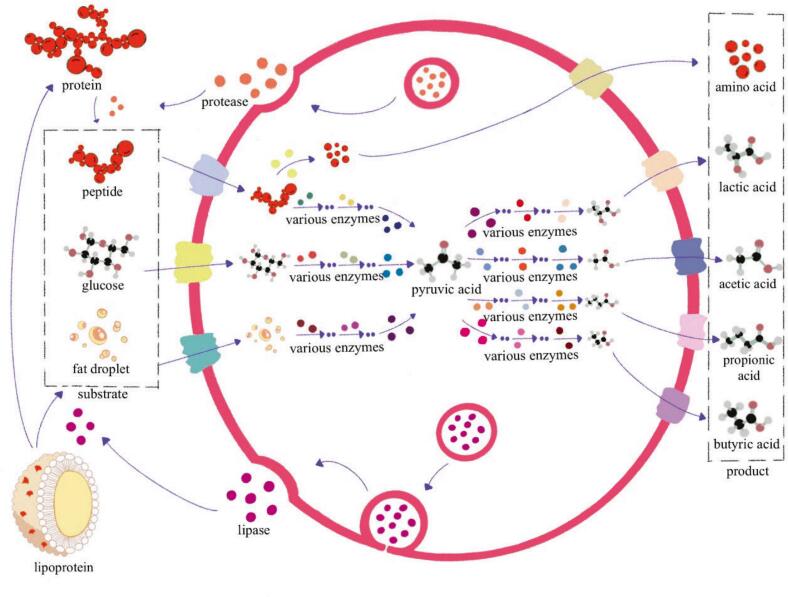
Transformations from egg-based substrates to products in LAB.

Material transformations during fermentation can be classified as either substrate consumption or product formation. Substrate consumption refers to the reduction of proteins, lipids, and exogenous carbon sources, while product formation covers metabolites such as acids, flavor compounds, and free amino acids (Fig. 1).

### 4.1 Substrate consumption

#### 4.1.1 Protein

Under LAB fermentation, egg-derived proteins in EW, EY, or whole liquid may be partially degraded. Jiang et al. reported a gradual decrease in total EW proteins during fermentation (native EW, ∼11% protein, no exogenous carbon source, initial pH 9.27, fermented with SST and LDB at 42 °C), reaching their lowest level at 9 h (Jiang et al., 2020). Jia et al. (2021) showed that *Lactobacillus* fermentation degraded ovalbumin, ovomucin, and ovomucoid into small peptides and free amino acids. Using LC-MS/MS, they identified peptide fragments derived from ovalbumin such as F.DVFKELKVHLA and F.DVFKELKVLA with molecular weights below 10 kDa, mainly from the N-terminal region of the protein, confirming that LAB proteolysis preferentially targets specific flexible domains of egg proteins. (Jia et al., 2021). The fermentation used 5% isolated EW proteins in defined medium with mixed carbohydrates (initial pH 5.5, 37 °C, four LAB strains). Yang et al. found that increasing the proportion of WE in a milk-based co-fermentation system (0–30% WE in milk with 8% sucrose, YO-MIX 883, 42 °C, pH 4.6) did not increase total protein correspondingly, indicating partial utilization of egg proteins by LAB (Yang et al., 2024). However, other studies detected no significant reduction in EW or EY proteins via SDS-PAGE before and after fermentation under the following conditions: (i) native EY (∼16% protein, no exogenous carbon source, natural pH 6.3, single *Lactobacillus* strain, 37 °C, 6 h) (Bie et al., 2025); (ii) undiluted EW (∼11% protein, no carbon source addition, initial pH adjusted to 9.20, complex LAB culture, 42 °C, 12–24 h) (Lv et al., 2024).

Compared with casein in milk, LAB exhibit lower proteolytic activity toward egg proteins due to: (1) higher final pH of egg-based fermentation (5.9–6.2 vs 4.5 in dairy) (Gundogan et al., 2023; Nahariah et al., 2015); and (2) higher molecular weights of egg proteins (∼35–76 kDa) and complex lipoprotein structures, which are less susceptible to LAB proteases (Bie et al., 2025; Lv et al., 2024). These intrinsic constraints underscore that egg matrices are inherently less favorable for LAB-driven proteolysis than dairy systems. Therefore, detectable hydrolysis in egg matrices is condition-dependent (Table 1), requiring exogenous carbon, low initial pH (≤5.5), EW or isolated protein, long fermentation (≥9 h), and/or mixed high-proteolytic strains.

**Table 1:** Key fermentation conditions and major outcomes of the representative experimental studies cited in this review.

Reference	Fermentation conditions	Final pH	Key outcomes measured	Direction of change	Analytical methods
(Jiang et al., 202)	Native EW (∼11% protein, no carbon source, initial pH 9.27) fermented with LDB + SST at 42 °C for 3–9 h	8.94	Total protein; glucose; ∼5 kDa peptides; FAA (Ala, Met, Pro); LAB abundance; free -SH; H₀; ζ-potential; secondary structure; apparent viscosity; FC; FS	Protein: ↓; Glucose: ↓; 5 kDa peptides: ↑; FAA (Ala, Met, Pro): ↑ then ↓; LAB abundance (Firmicutes): ↑; Free -SH: ↓; H₀: ↑ then ↓; ζ-potential: ↓; α-helix: ↑ then ↓; β-sheet: ↓ then ↑; β-turn: ↑; Apparent viscosity: ↓; FC: ↑ then ↓; FS: ↔	SDS-PAGE, HPLC-SEC; HPLC-RID; FAA: HPLC; 16S rDNA sequencing; Ellman's reagent; ANS fluorescence; Zeta potentiometer; FTIR; Rheometer; Dynamic Foam Analyzer
(Bie et al., 2025)	Native EY (∼16% protein, no carbon source, initial pH 6.3) fermented with LP, LR, LC, LA or LDB separately at 37 °C for 6 h.	5.0–5.3	pH; protein profiles; solubility; microstructure; volatile compounds; mayonnaise sensory acceptability	pH: ↓; Protein: ↔; Solubility: ↑; Microstructure: tight spherical → loose/porous; Volatiles: 3-methylbutanoic acid and 2-heptanone produced; Sensory: LA highest	pH meter; SDS-PAGE; centrifugation + freeze-drying; SEM; HS-SPME-GC–MS; Mayonnaise preparation + sensory evaluation (9-point hedonic scale)
(Gao et al., 2025)	EW (5% protein, 2% glucose) with enzymatic pre-hydrolysis, then fermented with LDB + SST at 42 °C for 12 h.	4.6–4.8	SCFAs; pH; volatile compounds; free amino acids; molecular weight distribution; particle size; sensory	SCFAs: ↑; pH: ↓; Volatiles: acetone, acetoin/diacetyl, acetic/butyric acids ↑; FAA: ↑; Low-MW components: ↑; High-MW components: ↓; Particle size: ↑; Fishy odor: ↓; Fermented aroma: ↑	pH meter; GC–MS; Amino acid analyzer/HPLC; HPLC-SEC; DLS; Sensory evaluation (20-point scale); Electronic nose
(Jia et al., 2022)	EW (∼11% protein, no carbon, pH 5.5 adjusted) fermented with LDB + SST at 37 °C for 3–9 h	Not known	Organic acids; volatile compounds; cake hardness; sensory odor score	Acetic, hexanoic, octanoic, nonanoic, decanoic acids: ↑; Nonanal, heptanal, benzaldehyde: major flavor contributors; 2,3-Pentanedione: ↑; Cake hardness: ↑; Odor score: ↑	HS-SPME-GC–MS; Texture analyzer; Sensory evaluation (odor score)
(Jia et al., 2024)	EW (∼11% protein, no carbon) fermented with SST at 37 °C for 4–12 h	Not known	Secondary structure; microstructure; moisture content; solubility; ζ-potential; particle size; FC; FS; H₀	β-sheets: ↓; α-helices: ↓; β-turns: ↑; Microstructure: spherical →more uniform; Moisture content: ↑; Solubility: ↑; ζ-potential: ↑; Particle size: ↓; FC: ↑; FS: ↑; H₀: ↓	FTIR; SEM; Oven drying; Centrifugation + freeze-drying; Zeta-sizer; Contact angle analyzer; Spectro-fluorophotometer
(Jia et al., 2021)	Isolated EW proteins (5% protein, with mixed carbohydrates, pH 5.5) fermented with LDB, LA, SST or BA at 37 °C for 3–9 h	4.8	Protein degradation; free amino groups; free -SH; H₀; ζ-potential; apparent viscosity; FC; FS	pH: ↓; Protein: OVA degraded to peptides; Free amino groups: ↑; Free -SH: OVA ↔; OVN ↑; OVM ↑; H₀: ↑then ↓; ζ-potential: ↑; Apparent viscosity: OVA ↓; OVN ↓; OVM ↑ then ↓; FC: ↑then ↓; OVN and OVM ↑; FS: OVA ↓; OVN ↑; OVM ↑	pH meter; OPA assay; LC-MS/MS; Ellman's reagent; ANS fluorescence; Zeta-sizer; Rheometer; Homogenizer
(Jia et al., 2025)	EY (∼16% protein, no carbon, pH 5.73) fermented with LR at 37 °C for 3.5 h	5.75	FAA; moisture content; solubility; EAI	FAA: ↑; Moisture content: ↑; Solubility: ↑; EAI: ↑	HPLC; Oven drying; Centrifugation + freeze-drying; Emulsion preparation + spectrophotometry
(Lv et al., 2024)	EW (8.1–11% protein, 1% glucose, pH 9.20) fermented with LDB and SST at 42 °C for 12–24 h	8.82–8.91	Microbial composition; pH; protein composition; H₀; tertiary structure; gel hardness; WHC; microstructure	pH: ↓; Protein: no change; H₀: ↑; Fluorescence intensity: ↓; Hardness (undiluted EW): ↓; Hardness (diluted EW): ↑; WHC: ↓; Microstructure: undiluted denser/homogeneous; diluted with glucose more evenly distributed aggregates	16S rRNA sequencing; pH meter; SDS-PAGE; ANS fluorescence; Fluorescence spectrophotometer; Texture analyzer; Centrifugation (WHC); SEM
(Tian et al., 2021)	EY (∼16% protein, no carbon, pH 6.09) fermented with LDB and SST at 42 °C for 3–9 h	5.49	Microbial composition; glucose level; solubility; free -SH; H₀; ζ-potential; secondary structure; thermal properties; apparent viscosity; EAI; ESI; emulsion microstructure	Firmicutes: ↑; Glucose: ↓; Solubility: ↑ then ↓; Free -SH: ↓; H₀: ↓; ζ-potential: ↑ then ↓; β-sheets: ↓ then ↑; α-helices: ↑ then ↓; β-turns: ↑; ΔH: ↓; Apparent viscosity: ↓; EAI: ↑; ESI: ↑ then ↓; Emulsion droplets: ↓ size, more uniform	16S rRNA sequencing; HPLC-RID; Centrifugation + freeze-drying (solubility); Ellman's reagent; ANS fluorescence; Zeta-sizer 2000; FTIR; DSC; Rheometer; UV spectrophotometry (EAI/ESI); Fluorescence microscope
(Yang et al., 2024)	WE in milk (3.3–6.6% protein, 8% sucrose) + heat pretreatment (65 °C, 20 min) fermented with LDB and SST at 42 °C for 7 h	4.6	Texture; WHC; rheological properties; volatile compounds; total lactic acid bacteria count; sensory evaluation	Hardness: ↑ then ↓; Consistency: ↑; Cohesiveness: ↑; WHC: ↑; Apparent viscosity: ↑; G′, G″: ↑; Volatiles: ↑; Total LAB count: ↑; Sensory overall acceptability: 5% WE addition highest	Texture analyzer; Centrifugation (WHC); Rheometer; Electronic nose; Plate count; Sensory evaluation (9-point hedonic scale, 20 panelists)
(Zang et al., 2023)	WE (4–5% protein, 11% sucrose, pH 6.0) pretreated at 100 °C, 40 min and homogenization, then fermented with LDB and SST at 42 °C	4.0–4.8	WHC; texture; rheology; particle size; ζ-potential; microstructure; protein composition; intermolecular forces; -SH; H₀	WHC: ↓; Hardness: ↑; Apparent viscosity: ↑; G′, G″: ↑; Particle size: ↑; Microstructure: porous →”hilly” morphology; H₀: ↑; Disulfide bonds: ↓; Free -SH: ↓; Total -SH: ↔; H₀: ↑; Protein hydrolysis: no significant changes	Centrifugation (WHC); Texture analyzer; Rheometer; Zeta-sizer Nano ZS90; SEM; SDS-PAGE; Ellman's reagent; ANS fluorescence

Note: * EW, Egg White; EY, Egg Yolk; WE, Whole Egg; LAB, Lactic Acid Bacteria; LDB, *Lactobacillus delbrueckii* subsp. *bulgaricus* (also referred to as LB, *Lactobacillus bulgaricus*); SST, *Streptococcus salivarius* subsp. *thermophilus*; LP, *Lactobacillus plantarum*; LR, *Lactobacillus reuteri*; LC, *Lactobacillus casei*; LA, *Lactobacillus acidophilus*; BA, *Bifidobacterium animalis*; SDS-PAGE, Sodium Dodecyl Sulfate–Polyacrylamide Gel Electrophoresis; HPLC-SEC, High-Performance Liquid Chromatography–Size Exclusion Chromatography; HPLC-RID, High-Performance Liquid Chromatography–Refractive Index Detector; FAA, Free Amino Acids; ANS, 1-Anilino-8-naphthalenesulfonate; FTIR, Fourier Transform Infrared Spectroscopy; FC, Foaming Capacity; FS, Foaming Stability; SEM, Scanning Electron Microscopy; HS-SPME-GC–MS, Headspace Solid-Phase Microextraction–Gas Chromatography–Mass Spectrometry; GC–MS, Gas Chromatography–Mass Spectrometry; DLS, Dynamic Light Scattering; OPA, o-Phthalaldehyde Assay; LC-MS/MS, Liquid Chromatography–Tandem Mass Spectrometry; EAI, Emulsifying Activity Index; ESI, Emulsifying Stability Index; DSC, Differential Scanning Calorimetry; WHC, Water-Holding Capacity; G′, Storage Modulus; G″, Loss Modulus; ΔH, Enthalpy Change; TPA, Texture Profile Analysis; OVA, Ovalbumin; OVN, Ovomucin; OVM, Ovomucoid; CDM, Chemically Defined Medium; BPB, Bromophenol Blue; DTNB, 5,5′-Dithiobis-(2-nitrobenzoic acid) (Ellman's Reagent); H₀, Surface Hydrophobicity; -SH, Free Sulfhydryl Group

#### 4.1.2 Lipid

LAB may exert certain degradative effects on egg-derived lipids in EY or WE. All studies to date report a decrease in lipid or phospholipid content after fermentation, although the extent of degradation is relatively limited. Fermentation with *Lactobacillus acidophilus*, *Lactobacillus casei* and *Lactobacillus plantarum* significantly reduced fat and phospholipid content in EY (Li et al., 2024). Yang et al. reported no corresponding gradient in total fat content with increasing WE proportion in a milk-based co-fermentation system, indicating partial lipid utilization (Yang et al., 2024). Flavor compounds in fermented EY originate mainly from lipid hydrolysis and peroxidation by LAB (Bie et al., 2025; Tang et al., 2024). Protease treatment has also been shown to reduce fat and cholesterol content (Gu et al., 2021). However, most EY lipids exist as lipoprotein complexes, limiting fermentation efficiency. Enhancing lipid fermentation may require overexpression of lipo-proteolytic enzymes in LAB or exogenous enzyme supplementation.

#### 4.1.3 Exogenous carbon source

Liquid egg matrices lack sufficient carbon sources, so glucose is commonly added and consumed during fermentation. Glucose levels gradually declined in EY and EW as fermentation progressed (Jiang et al., 2020; Tian et al., 2021), as LAB metabolize glucose via glycolysis for energy and acid production. This inherent carbon deficiency is a major constraint for LAB fermentation in egg systems, as LAB cannot sustain robust growth and acid production without exogenous carbohydrate supplementation. LAB growth depends on protein, lipid, and exogenous carbon content, with initial pH also significantly affecting growth. Zang et al. optimized these parameters based on milk's nutritional profile, adjusting initial pH to 6.5 (Zang, Pan, et al., 2023; Zang, You et al., 2023). Thus, the addition of exogenous carbon sources is not merely beneficial but essential for achieving effective fermentation in egg-based systems.

### 4.2 Product formation

#### 4.2.1 Small acidic compounds

During fermentation, substrates are converted into low-molecular-weight acidic compounds, flavor substances, and free amino acids. Acidic compounds contribute to the pH decline (Wang et al., 2022). The pH of WE dropped from 6.5 to 6.8 to 5.5–5.8 (Zang, You, et al., 2023; Zang, Pan et al., 2023), the EY pH from 6.3 to 5.3–5.5 (Bie et al., 2025), and the EW pH to 4.8 (Jia et al., 2021).

This decline is attributed to the production of organic acids by LAB during fermentation. These acids include SCFAs such as formic, acetic, propionic, and butyric acids, as well as branched-chain and medium-chain fatty acids. For instance, LAB fermentation elevated acetic, butyric, and isobutyric acids in EW (Gao et al., 2025). Similarly, after 3 h of fermentation with SST and LDB, EW produced notable amounts of hexanoic, octanoic, nonanoic, and decanoic acids (Jia et al., 2022). In EY, fermentation by LDB generated high levels of 3-methylbutanoic acid (Bie et al., 2025). Notably, among these organic acids, the SCFAs have been recognized for their potential gut–brain axis modulatory effects in vivo (Kim et al., 2024), although this has not been demonstrated in LAB-fermented egg systems.

#### 4.2.2 Flavor compounds

LAB fermentation converts substrates into diverse flavor compounds. Electronic nose analysis showed that yogurt from the milk-WE co-fermentation system exhibited higher sensor responses to methyl species and organic aromatic sulfides, and sensory evaluation confirmed that the 5% WE addition group received the highest overall acceptability scores (Yang et al., 2024). GC–MS analysis revealed that fermented EW exhibited aromatic flavors from acetone, buttery notes from acetoin/diacetyl, and acidic notes from acetic/butyric acids, while sensory evaluation demonstrated significantly reduced fishy odor (scores <5/20) and enhanced fermented aroma in the fermented samples (Gao et al., 2025). Aldehydes (nonanal, heptanal, benzaldehyde) and ketones (2,3-pentanedione) were major contributors to flavors in cakes made with fermented EW, and sensory evaluation confirmed that the 9 h-fermentation group significantly improved odor scores (Jia et al., 2022). In EY, 3-methylbutanoic acid and 2-heptanone imparted acidic and fruity flavors, respectively; sensory evaluation further showed that mayonnaise made with L. *acidophilus*-fermented EY received the highest flavor and overall acceptability scores, whereas L. *reuteri*-fermented EY yielded lower scores, indicating that strain selection is critical for consumer acceptability (Bie et al., 2025). In summary, EW and EY support diverse flavor compounds such as aldehydes, ketones and acids that are acceptable to consumers with appropriate strains and conditions.

#### 4.2.3 Free amino acids

LAB fermentation releases free amino acids from egg-derived matrices. Fermented EW powder (N-4, the n-4 sample after subsequent LAB fermentation) contained higher levels of free serine, threonine, alanine, cysteine, methionine, phenylalanine, and leucine than its unfermented counterpart (n-4, the egg white sample after enzymatic hydrolysis only). (Gao et al., 2025). Similarly, a significant increase in alanine, methionine, and proline was observed in fermented EW powder (Jiang et al., 2020). In EY, fermentation with *Lactobacillus acidophilus*, *Lactobacillus casei* and *Lactobacillus plantarum* markedly raised the free amino acid content (Li et al., 2024). *Limosilactobacillus reuteri* and L. *rhamnosus* increased free amino acids by 32% and 56%, respectively (Jia et al., 2025). However, prolonged fermentation may lead to a decline in certain free amino acids (Jiang et al., 2020). LAB proteases hydrolyze egg proteins to generate free amino acids, some of which are subsequently utilized for further metabolism (Sun et al., 2019). In summary, LAB fermentation releases free amino acids from both EW and EY, with the extent depending on strain, fermentation time, and substrate composition.

## 5 Effects and mechanisms of egg-derived matrices on LAB growth

LAB growth in egg-derived matrices is well documented. Significant increases in viable counts were reported for *L. plantarum* in EW (Nahariah et al., 2015), *Streptococcus salivarius* subsp. *thermophilus* (SST) and *Lactobacillus delbrueckii* subsp. *bulgaricus* (LDB) in EY (Tian et al., 2021) and in EW (Jiang et al., 2020; Lv et al., 2024). Different egg components selectively promote distinct bacterial genera, suggesting specific fractions favor particular strains. However, LAB exhibit slower growth in egg-based systems compared with dairy (4–6 h). This is attributed to: (1) the lack of readily available carbohydrates; (2) lysozyme requiring adaptation; (3) the high molecular weight and structural complexity of egg proteins demanding greater protease secretion; and (4) the initially alkaline pH of fresh EW (typically 7.6–9.7, depending on storage conditions), which is suboptimal for LAB growth and acid production. These constraints collectively indicate that egg liquid is not an ideal fermentation substrate for LAB, and successful fermentation requires careful optimization of exogenous carbon supplementation, pH control, and strain selection to overcome these limitations.

## 6 Effects of LAB fermentation on structural changes of egg-derived protein

**Fig. 2:**
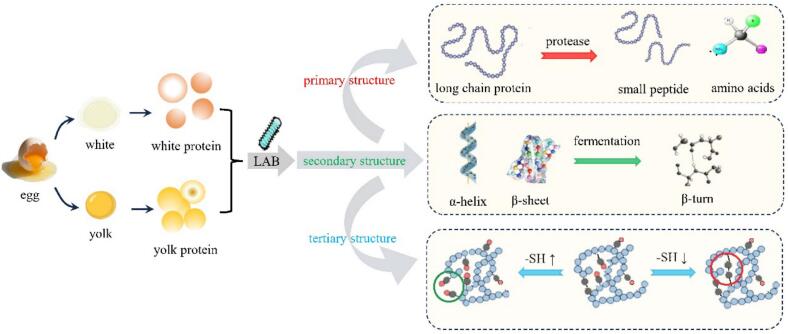
LAB-induced primary, secondary and tertiary structural changes of white and yolk proteins.

Structural changes are categorized into apparent surface morphology via scanning electron microscopy (SEM) and molecular (primary, secondary, tertiary) levels (Fig. 2).

### 6.1 Apparent structural changes

Fermentation alters the apparent structure of WE, EW, and EY. In WE, fermentation induces a transition from a porous network to a dense, loosely gelled “hilly” morphology as aggregation progresses (Chang et al., 2024; Zang, Pan, et al., 2023; Zang, Zhang et al., 2023). In EW powder, native spherical, smooth, non-uniform particles develop concave structures with reduced size and improved uniformity after fermentation (Jia et al., 2024; Lv et al., 2024). Similarly, unfermented EY displays tightly packed spherical structures, which become looser and more porous after fermentation (Bie et al., 2025; Li et al., 2024), with cavities and improved uniformity (Jia et al., 2025). This open structure provides more effective binding sites for interfacial adsorption (Liu et al., 2024). In summary, LAB modify the microstructure of WE, EW, and EY via hydrolysis, aggregation, and cross-linking, resulting in smaller, more uniform particles and porous or dense gel networks.

### 6.2 Molecular structural changes

#### 6.2.1 Changes in primary structure

As detailed in Section 4.1.1, LAB proteases hydrolyze egg proteins condition-dependently, generating low-molecular-weight peptides and free amino acids under optimal conditions. Compared with unfermented controls (p-4 and n + p-4, samples after enzymatic hydrolysis only), fermented EW (P-4 and N + P-4, the corresponding samples after subsequent LAB fermentation) contained a higher proportion of low-molecular-weight components (1000–5000 Da) and a lower proportion of high-molecular-weight components (>10,000 Da) (Gao et al., 2025). After 9 h of fermentation, ovalbumin (∼40 kDa) decreased from 72.54% to 70.77%, while ∼5 kDa peptides increased from 2.02% to 2.74% (Jiang et al., 2020). Additionally, *Lactobacilli* fermentation can degrade ovalbumin, ovomucin, and ovoglobulin into free amino acids (Jia et al., 2021). However, no significant changes occur under suboptimal conditions (Bie et al., 2025; Lv et al., 2024; J. W. Zang, H. T. You, et al., 2023). The limited proteolytic capacity of LAB in egg matrices is due to higher final pH, restricted protease activity, and the inherent complexity of egg proteins. Synergistic use of exogenous proteases with LAB may enhance protein breakdown and utilization.

#### 6.2.2 Changes in secondary structure

Fermentation also alters the secondary structure of egg-derived proteins, affecting the content of α-helices, β-sheets, and β-turns (Wang et al., 2022). For example, fermentation by L. *rhamnosus* increased the proportion of β-sheets and β-turns while decreasing α-helices in EY. Conversely, Tian et al. observed a decrease in β-sheets and increases in α-helices and β-turns in EY after 6 h of LAB fermentation (Tian et al., 2021). For EW, fermentation during the exponential growth phase reduced the proportions of β-sheets and α-helices while increasing β-turns (Jia et al., 2024; Jiang et al., 2020). In summary, although the respective effects on α-helix and β-sheet content may vary, fermentation generally reduces their combined proportion while increasing the proportion of β-turns.

α-helices and β-sheets contribute to stability through strong intramolecular non-covalent interactions, while β-turns confer greater molecular flexibility due to weaker intramolecular forces (Alavi, Emam-Djomeh, et al., 2020; Zhang et al., 2023). It can be inferred that LAB fermentation promotes the conversion of α-helices or β-sheets into β-turns, thereby reducing protein stability and increasing conformational flexibility. This structural shift reduces stability, increases flexibility, and may enhance foaming capacity and bioactive interactions (Alavi, Emam-Djomeh, et al., 2020; Duan et al., 2017).

#### 6.2.3 Changes in tertiary structure

The impact on free sulfhydryl (-SH) content is variable (Li et al., 2019; Xu et al., 2018). Some studies report a continuous decrease in free sulfhydryl groups during fermentation. For instance, Jiang et al. observed a continuous decline in free sulfhydryl content in fermented EW, reaching a minimum at 9 h with a 17.9% reduction (Jiang et al., 2020). Similarly, Zang et al. reported a 34% decrease in free sulfhydryl content in fermented WE after 10 h (J. Zang et al., 2023), and Tian et al. noted a 42% drop in fermented EY by the end of fermentation (Tian et al., 2021). Conversely, other studies report an increase. Zang et al. found an ∼20% rise in free sulfhydryl content in WE after 10 h of fermentation (J. W. Zang, H. T. You, et al., 2023). Jia et al., fermenting isolated EW fractions (ovalbumin, ovomucin, ovomucoid) with *Lactobacilli*, observed an increase only in ovalbumin and ovomucoid (Jia et al., 2021). These results indicate that the effect of LAB fermentation on free sulfhydryl content in egg proteins is variable. This variability depends on three factors. First, inherent -SH content differs among egg proteins (ovalbumin> ovomucoid> ovomucin) (Jia et al., 2021; Li-Chan & Kim, 2008; Omana et al., 2010). Second, higher-order structure can shield -SH groups (Sheng et al., 2019). Third, LAB metabolites participate in redox reactions with -SH/disulfide bonds (Lagrain et al., 2007; Xu et al., 2018).

Consequently, the change in free sulfhydryl content depends on the balance between LAB-induced reduction and oxidation. An increase is typically observed when proteolytic exposure (Sections 6.2.1) dominates, such as in isolated ovalbumin or ovomucoid under fermentations that promote protein unfolding. A decrease occurs when oxidative cross-linking prevails, which is more common in WE, EY, or under conditions that favor disulfide bond formation. Thus, the direction of change is condition-dependent and should be interpreted in light of the specific protein substrate, fermentation conditions, and the prevailing redox environment.

## 7 Effects and mechanisms of LAB fermentation on PP of egg

This section details fermentation-induced changes in aqueous solution characteristics, texture, rheology, ζ-potential, particle size, hydrophobicity, emulsifying capacity, and foaming properties, with mechanisms cross-referenced to Section 6 where applicable.

### 7.1 Aqueous solution characteristics

Fermentation can alter the moisture content of spray-dried powders derived from EW or EY. Fermentation by L. *reuteri* or L. *rhamnosus* increased the moisture content of spray-dried EY powder from 2.31% to 2.68% and 2.57%, respectively (Jia et al., 2025). Similarly, fermentation raised the moisture content of spray-dried EW powder from an initial 4.93% to 5.16–5.81% (Jia et al., 2024), attributed to reduced particle size and increased water-binding sites (Abreha et al., 2021; Jia et al., 2025), while free water in liquid systems decreases as a result of protein aggregation and gel network formation, which restrict water mobility and convert free water into bound water (J. W. Zang, H. T. You, et al., 2023).

The solubility of EW and EY consistently improves. Fermentation of EW by L. *plantarum* initially increased soluble protein content, peaking at 18 h followed by a decline (Nahariah et al., 2015). Fermentation significantly improves the solubility of EW powder (Jia et al., 2024). Fermentation with various LAB strains enhanced EY protein solubility by 9.4%–31% (Bie et al., 2025). Fermentation by *L. reuteri* or *L. rhamnosus* increased the solubility of EY powder from 13.92% to 23.08% and 17.53%, respectively (Jia et al., 2025). Correspondingly, a rise in EY solubility from 15.5% to 25.5% was reported after 3 h of fermentation (Tian et al., 2021), consistent with findings by Li et al. (Li et al., 2024). This improvement is attributed to protease-induced reduction in molecular weight and reorientation of hydrophobic groups (Bao et al., 2017; Gu et al., 2021), mechanisms detailed in Section 6.2.1.

Fermentation also affects the water-holding capacity (WHC) of egg-based systems. Zang et al. observed a continuous decrease in the WHC of liquid egg as fermentation progressed (J. Zang et al., 2023; J. W. Zang, H. T. You, et al., 2023), a trend corroborated by Lv et al. for EW (Lv et al., 2024). WHC reflects water-protein binding, and fermentation weakens the gel network, reducing WHC. This decline is linked to three factors: (i) reduced disulfide bonds (Sections 6.2.3) and hydrophobic interactions that weaken gel strength (Li et al., 2018); (ii) enhanced protein aggregation, which correlates with lower WHC (Lv et al., 2024; J. W. Zang, H. T. You, et al., 2023); and (iii) lower protein concentration yielding weaker gels (Iwashita et al., 2015; Yang et al., 2024). In summary, fermentation reduces particle size and molecular weight to enhance solubility, but decreases WHC via weakened gel networks and protein aggregation.

### 7.2 Texture characteristics

Fermentation can alter textural properties such as hardness and consistency of liquid egg, but the reported effects vary. Under moderate dilution (protein 4–8%), fermentation typically increases gel hardness. For example, adding 5% WE to milk (final protein ∼3.3%) in a co-fermentation system significantly increased hardness, consistency, and cohesiveness (Yang et al., 2024). Similarly, fermentation for 4–10 h markedly enhanced the hardness of WE under comparable diluted conditions (final protein 4–5%) (J. Zang et al., 2023), and diluted EW (protein ∼8%) showed increased hardness during fermentation (Lv et al., 2024). Cakes prepared with fermented EW also exhibited higher hardness (Jia et al., 2022).

Conversely, under conditions of high protein concentration (≥11%, undiluted EW or high egg-to-milk ratios), fermentation often decreases hardness. Specifically, increasing the proportion of WE in milk to >10% in the co-fermentation system lowered its hardness (Yang et al., 2024), and the hardness of undiluted EW decreased significantly as fermentation progressed (protein ∼11%) (Lv et al., 2024). Thus, fermentation effects on texture are condition-dependent, governed primarily by initial protein concentration or dilution level. Moderate protein (≤4–8%) favors a well-structured gel network, whereas excessive protein (≥11%) may promote over-aggregation or proteolytic weakening, reducing hardness. Dilution degree should therefore be considered a key factor in product development.

The effect of fermentation on hardness reflects its modulation of protein gel network strength. Gel strength depends on the balance between electrostatic repulsion and hydrophobic interactions among protein molecules (Huang et al., 2019). The former is largely influenced by the final pH (Chen et al., 2022), while the latter is closely associated with the surface hydrophobicity of the egg proteins.

In summary, texture changes appear to depend on protein concentration, with moderate dilution (approximately 4–8% protein) tending to increase hardness, while higher concentrations (≥11%) often reduce it. However, these values should be interpreted as tentative cross-study trends rather than fixed thresholds, because the compared studies differ in matrix composition (EW vs. EY vs. WE), strain selection, sugar supplementation, heat pretreatment, initial pH, fermentation time, and analytical conditions. The observed pattern nonetheless provides a useful empirical guide for product development, but precise threshold values require further validation under controlled conditions.

### 7.3 Rheological characteristics

Fermentation also modifies the rheological properties of EW, EY, or WE, but its effects are inconsistent. Some studies report a positive correlation between fermentation time and rheology. For instance, Zang et al. observed a significant increase in the apparent viscosity of WE (J. W. Zang, H. T. You, et al., 2023). During 0–10 h fermentation, the apparent viscosity at 0.1 s^−1^ gradually increased (J. Zang et al., 2023). In contrast, other studies indicate a negative correlation: longer fermentation resulted in lower apparent viscosity for EW (Jiang et al., 2020) and EY (Tian et al., 2021).

Although these observations suggest a tentative cross-study pattern, direct quantitative comparisons should be made with caution, as the compared studies differ in matrix composition, strain selection, sugar supplementation, heat pretreatment, initial pH, fermentation time, and analytical conditions (Table 1). A potential pattern emerges when comparing these effects to dairy systems. Fermentation consistently enhances milk viscosity, whereas its impact on egg matrices varies, likely due to protein concentration. Milk (∼3.5% protein) shows a positive viscosity-fermentation time correlation (Maharani et al., 2020; Marete et al., 2024), whereas native EW or EY (11–16% protein) exhibits a negative correlation (Jiang et al., 2020; Tian et al., 2021). Interestingly, when an egg-based solution is diluted to ∼4.5% protein (matching milk's profile), its viscosity also positively correlates with fermentation time (J. Zang et al., 2023; J. W. Zang, H. T. You, et al., 2023). These results suggest that the viscosity-fermentation time relationship depends on protein concentration, and an optimal protein content is crucial for developing egg-based products with desirable rheological properties. However, the specific concentration values (∼4.5% vs 11–16%) should be interpreted as tentative cross-study trends rather than established cut-off values, given the substantial inter-study variability in experimental conditions and analytical protocols.

In conclusion, the influence of protein concentration on viscosity is fundamentally linked to how LAB interact with the egg matrix. At suitable protein concentrations, LAB growth is more favorable. First, LAB proteolysis (Section 6.2.1) generates low-molecular-weight peptides and amino acids. The exposure of additional amino hydrogens (N—H) and hydroxyl oxygens (O—H) from these small molecules enhances hydrogen bonding between peptides, as well as between peptides and exopolysaccharides, thereby contributing to increased viscosity (Zhou et al., 2019). Second, low pH (as detailed in Section 4.2.1) reduces electrostatic repulsion and promotes hydrophobic aggregation, which increases viscosity and imparts specific rheological behavior (Chen et al., 2022; Huang et al., 2019). Third, higher water content facilitates interactions between exposed hydrophobic regions. While this mechanistic framework provides a coherent explanation for the observed trends, systematic studies under standardized conditions are needed to define precise concentration thresholds and validate the proposed mechanisms.

### 7.4 ζ-potential and particle size

A higher absolute ζ-potential value and a smaller particle size typically indicate greater stability (Li & Chiang, 2012; J. W. Zang, H. T. You, et al., 2023). LAB fermentation influences both parameters, though reported effects vary. Some studies report reduced absolute ζ-potential or increased particle size. For example, EW protein particles shifted from 1 to 10 μm to 1000 μm after fermentation (Gao et al., 2025), Zang et al. reported particle size of EW increased from 25 nm to 1200 nm (J. Zang et al., 2023), and the absolute value of ζ-potential gradually decreased during fermentation (Jiang et al., 2020). Conversely, other studies report increased absolute ζ-potential or reduced particle size. Jia et al. found that fermentation significantly increased the absolute ζ-potential of ovalbumin, ovomucin, and ovomucoid (Jia et al., 2021). Jia et al. reported reduced particle size of EW powder from 2.59 μm to 0.76–1.69 μm (Jia et al., 2024). Additionally, some findings show a trend where the absolute value of ζ-potential initially rises before declining during fermentation (Tian et al., 2021).

These contradictions arise from the balance between two competing processes: (i) proteolytic exposure of charged groups (Sections 6.2.1) and (ii) pH-driven charge neutralization with hydrophobic aggregation. The absolute value of ζ-potential increases when proteolysis dominates. pH stays well above pI and hydrolysis exposes charged residues, typical of isolated proteins or highly proteolytic conditions (Jia et al., 2021; Jia et al., 2024). Conversely, it decreases when pH-driven neutralization and hydrophobic aggregation prevail, as pH approaches pI, typical in complex matrices (initial pH ≥9). An initial rise then decline reflects early exposure dominance followed by charge aggregation (Tian et al., 2021).

Unlike the effect of pure proteases on particle size (Bao et al., 2017; Gu et al., 2021), the impact of LAB fermentation on the absolute value of ζ-potential and particle size of egg proteins in emulsions is determined by multiple concurrent factors. First, proteolytic (Section 6.2.1) exposure of hydrophilic groups alters the surface charge of protein molecules (Fu et al., 2020; Tian et al., 2021). Second, pH reduction from acid production (Section 4.2.1) modifies the charge status of these groups relative to the isoelectric point, thereby influencing ζ-potential (Li et al., 2024). Third, fermentation-induced structural modifications (6.2.2, 6.2.3) alter hydrophobic interaction zones and aggregation behavior, affecting particle size distribution (Alavi, Tian, et al., 2020). In summary, the absolute value of ζ-potential increases with proteolytic exposure but decreases with pH-driven aggregation, which also increases particle size.

### 7.5 Hydrophobicity

LAB fermentation also influences the surface hydrophobicity (H₀) of egg-derived proteins, with reported effects varying. Some studies report positive effects: fermented EW showed higher H₀ than unfermented (Chang et al., 2024), WE exhibited an 81.1% increase (J. Zang et al., 2023), and EW confirmed a 31.0% rise after 12 h (Lv et al., 2024). In contrast, others indicate a negative impact: EY H₀ decreased from 380.66 to 111.82, 57.17, and 20.29 after 3, 6, and 9 h of fermentation, respectively (Tian et al., 2021). Some research also shows an initial increase followed by a decrease in H₀ of ovalbumin, ovomucin, and ovomucoid during fermentation (Jia et al., 2021; Jiang et al., 2020).

H₀ is determined by two factors. First, the proportion of hydrophobic amino acids in the primary structure defines its maximum hydrophobic potential; ovalbumin (∼50% hydrophobic amino acids) possesses considerable hydrophobic potential among EW proteins (Visentini et al., 2017). Second, not all hydrophobic sites are surface-exposed; some spontaneously form internal hydrophobic clusters to minimize free energy. This process is facilitated by the high spatial flexibility of covalent bonds (Horne, 2006).

In summary, the net change in H₀ is governed by three concurrent mechanisms. First, proteolytic exposure of hydrophobic sites (Section 6.2.1) increases H₀ (Zhang et al., 2019). Second, disulfide bond formation (Section 6.2.3) can refold proteins, burying hydrophobic residues and decreasing H₀ (Ekezie et al., 2018; Singh et al., 2018), as discussed in Section 6.2.3. Third, acid production (Section 4.2.1) lowers pH toward the isoelectric point (∼4.5–5.0) (Section 7.4). At this pH, charge neutralization promotes hydrophobic patch aggregation, reducing H₀ (Xu et al., 2018). Thus, H₀ increases when proteolytic exposure dominates and decreases when pH-driven aggregation prevails. It shows an initial rise followed by a decline when exposure temporarily outpaces aggregation before pH falls sufficiently. The direction of change is therefore condition-dependent, governed by substrate composition, carbon source availability, initial pH, and fermentation time.

### 7.6 Emulsifying characteristics

EY is a primary focus for emulsification studies due to its excellent capacity to dissolve oil and water phases. Emulsifying properties are characterized by two indices: emulsifying activity index (EAI), related to the kinetic ability to cover the oil/water interface; and emulsifying stability index (ESI), related to the ability to resist droplet coalescence. LAB fermentation affects both EAI and ESI of EY, though reported effects vary. Tian et al. reported that fermentation for 3, 6, and 9 h increased EAI from 9.07 to 19.55, 23.40, and 24.61 m^2^/g, respectively, while ESI initially rose from 0.18 to 0.33 min, then decreased to 0.15 and 0.155 min (Tian et al., 2021). Similarly, Jia et al. observed significant EAI increases from 3.83 to 4.29 and 4.10 m^2^/g (Jia et al., 2025). Conversely, Li et al. found that fermentation significantly decreased both EAI and ESI of EY (Li et al., 2024).

Importantly, changes in EAI and ESI are not directly caused by fermentation conditions, but by fermentation-induced alterations in EY protein properties. These include solubility, H₀/hydrophilicity balance, secondary/tertiary structure, ζ-potential, and droplet size distribution. These factors collectively determine EAI and ESI, accounting for the discrepancies among studies.

EAI is influenced by three main factors: (i) protein solubility (Section 7.1), which facilitates oil–water mixing (Deng et al., 2011); (ii) H₀/hydrophilicity balance (Section 7.5), crucial for emulsion type (Castellani et al., 2006; Delahaije et al., 2015); and (iii) β-turn content (Section 6.2.2), which enhances molecular flexibility for improved emulsification (Fu et al., 2020; Jia et al., 2025).

ESI is affected by four key factors: (i) disulfide bond content (Section 6.2.3), strengthening interfacial film mechanical strength (Wu et al., 2011); (ii) absolute ζ-potential (Section 7.4), increasing electrostatic repulsion between droplets (Li et al., 2024); (iii) hydrophilic/hydrophobic balance, resisting protein aggregation (Nishinari et al., 2014); and (iv) droplet size, with smaller sizes leading to lower aggregation rates and greater stability. In summary, changes in EAI and ESI are governed by solubility, secondary structure, tertiary structure, H₀, ζ-potential, and droplet size. The direction depends on fermentation conditions and strains.

### 7.7 Foaming characteristics

EW proteins are notable for their excellent foaming properties due to their ease of adsorption at the air–water interface. Foaming performance is characterized by foaming capacity (FC), the ability to incorporate air, and foaming stability (FS), the ability to maintain the foam. LAB fermentation influences both FC and FS, though effects vary. Jia et al. found that appropriate fermentation significantly improved FC and FS of EW proteins, with component-dependent effects on FS (ovalbumin, ovomucin, and ovomucoid) (Jia et al., 2021; Jia et al., 2024). Jiang et al. also reported a significant increase in FC with fermentation, while FS showed no significant change (Jiang et al., 2020). Importantly, the variation in foaming properties is not directly determined by fermentation conditions, but by fermentation-induced alterations in protein properties. These include protein type, solubility, secondary structure, H₀, electrostatic repulsion, and particle size, which collectively account for study discrepancies.

FC is related to the rate of gas dispersion within the protein matrix. The impact of fermentation on FC is associated with five factors. First, the protein type greatly influences initial foaming capacity, with globular proteins like ovalbumin in EW exhibiting strong foaming ability (Foegeding et al., 2006; Li et al., 2019). Second, increased protein solubility allows for more frequent contact between gas and dissolved proteins (Section 7.1) (Bao et al., 2017; Zhang et al., 2023). Third, modifications to the protein's secondary structure (Section 6.2.2) enhance molecular flexibility, facilitating unfolding at the air–water interface (Duan et al., 2017). Fourth, increased H₀ (Section 7.5) improves interaction with the gas phase (Carpena et al., 2017). Fifth, altered electrostatic repulsion (Section 7.4) affects bubble formation rate (Xiong et al., 2016).

FS after bubble formation is influenced by the drainage rate of the liquid film between bubbles. Fermentation affects FS through three primary mechanisms. First, it can improve the viscoelasticity of the protein (Section 6.2.2), slowing down gravitational drainage and enhancing stability (Dabestani & Yeganehzad, 2019). Second, reduced electrostatic repulsion (Section 7.4) may allow bubbles to coalesce more easily, decreasing stability (Sheng et al., 2019). Third, a reduction in particle size (Section 7.4) can more effectively stabilize the air–water interface (Jafari et al., 2022). In summary, FC and FS are governed by solubility, structure, H₀, electrostatic repulsion, and particle size.

**Fig. 3:**
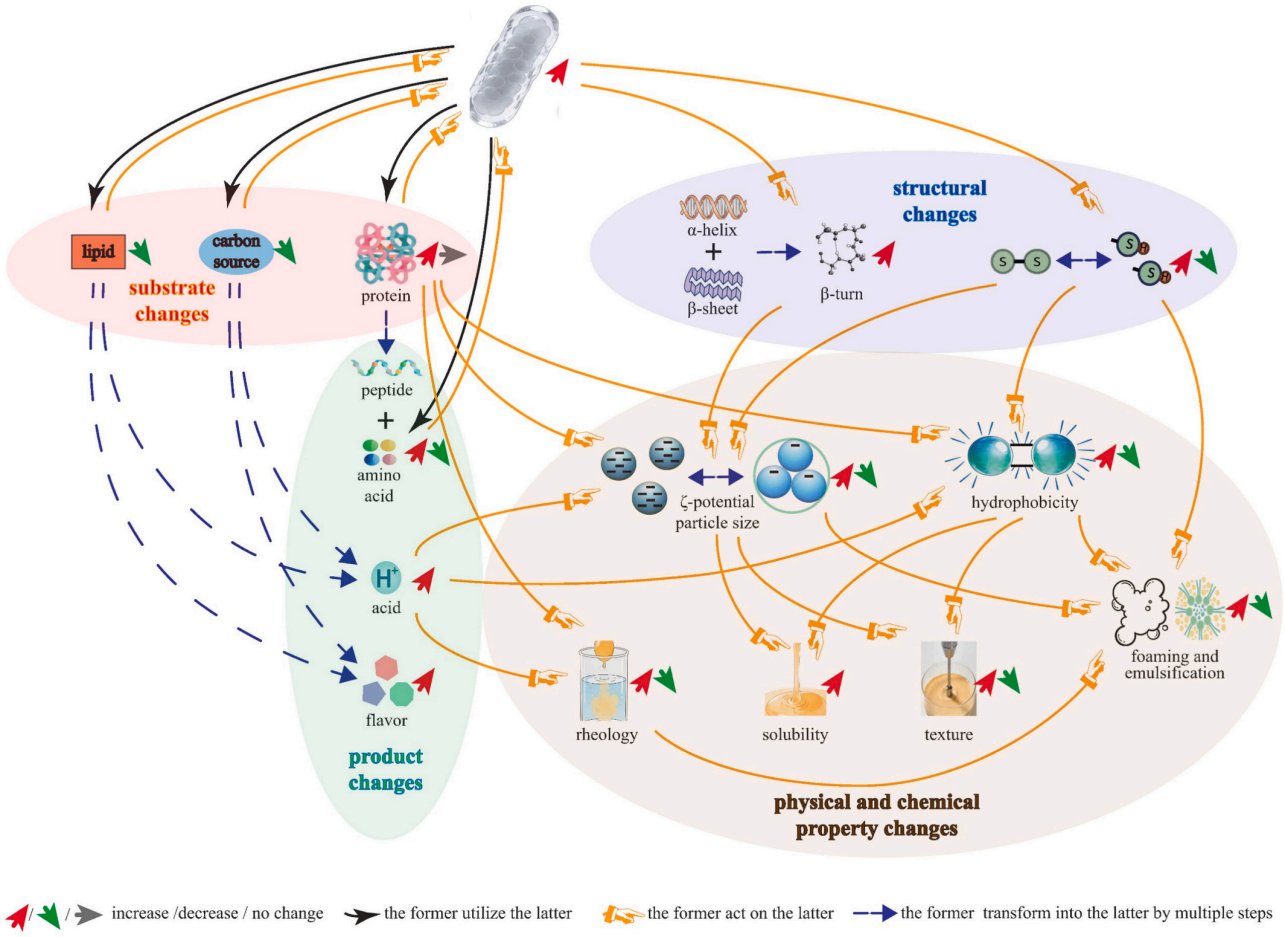
Conceptual framework illustrating the interconnections among substrate transformations, protein structural modifications, and physicochemical/functional properties of egg products during LAB fermentation.

In this work, we propose a coherent conceptual framework linking substrate changes, structural modifications, and PP of egg products during LAB fermentation (Fig. 3). Under fermentation, egg proteins, lipids, and exogenous carbon sources are acted upon by LAB, leading to their partial conversion into small peptides, amino acids, flavor compounds, and small organic acids. In turn, these substrates promote LAB growth and abundance. Concurrently, LAB fermentation induces secondary and tertiary structural alterations in egg proteins, including changes in β-turns and free sulfhydryl groups. These material and structural changes further influence multiple PP such as solubility, hydrophobicity, ζ-potential, particle size and rheology, either positively or negatively, depending on the extent and pathway of modification. Ultimately, the combined effects of structural and PP collectively determine the emulsifying and foaming properties of egg products. This framework reconciles previously discrepant findings and provides a mechanistic basis for understanding how fermentation conditions affect functional properties through direct changes in protein structure and composition.

## 8 Limitations and applications of fermented egg liquid

Before discussing specific research gaps, it is important to recognize the inherent limitations of egg liquid as a fermentation substrate for LAB. Unlike dairy systems, egg liquid presents multiple constraints that collectively hinder LAB growth and metabolic activity: (1) insufficient fermentable carbohydrates, requiring exogenous supplementation; (2) an initially alkaline pH in fresh EW, which is suboptimal for LAB growth and necessitates pH adjustment; (3) the presence of antimicrobial components such as lysozyme in EW, which may inhibit LAB proliferation; (4) high molecular weights of egg proteins and complex lipoprotein structures, which are poorly susceptible to LAB proteases and lipases; and (5) a higher final fermentation pH (5.9–6.2) compared with dairy (pH ∼4.5), which limits proteolytic activity. These constraints collectively indicate that egg liquid is not an ideal fermentation substrate for LAB, and successful fermentation requires careful optimization of exogenous carbon supplementation, pH control, strain selection, and fermentation conditions.

### 8.1 Limited exploration of bioactive potential

Based on the existing research outlined above, the current limitations in studying the LAB–egg matrix system are evident. Most studies focus on the impact of LAB on nutrient profiles, molecular structures, and PP of the matrix, while the health-promoting properties of egg-based substrates have been scarcely explored. Consequently, our research group intends to focus subsequent investigations on whether LAB can utilize egg-derived matrices to produce bioactive compounds such as gamma-aminobutyric acid (GABA), bacteriocins, and SCFAs. This will help unlock the health-promoting potential of egg-based substrates for applications in healthcare and gut microbiota modulation.

### 8.2 Uninvestigated bidirectional regulation

Research has predominantly emphasized the positive effects of LAB on egg matrices, whereas the reverse regulatory influence of egg matrices on LAB physiology remains largely uninvestigated. Therefore, we plan to prioritize studying the transcriptional regulation of LAB by egg-derived components. Further research will delve into the regulatory mechanisms governing the synthesis of GABA, bacteriocins, and SCFAs within these matrices. This approach aims to elucidate the bidirectional regulatory mechanisms between egg matrix components and LAB, laying a foundation for future exploration of their potential applications in functional food development and gut health research.

### 8.3 Potential safety risks and quality defects of LAB-fermented egg liquid

While LAB fermentation offers promising functional improvements, it may also introduce potential safety risks and quality defects that have not been thoroughly investigated in egg matrices.

A primary concern is the possible accumulation of biogenic amines (BAs), particularly histamine and tyramine, produced by microbial decarboxylation of free amino acids (Linares et al., 2011). This risk is well documented in other high-protein fermented foods (Park et al., 2019). However, no study to date has specifically monitored BA production during LAB fermentation of egg liquid, leaving a critical knowledge gap regarding unsafe fermentation conditions.

Another concern involves pathogen and spoilage control. Eggs are highly nutritious and susceptible to microbial contamination, with *Salmonella* being a major safety risk in egg products. Although LAB may inhibit *Salmonella* through acidification and bacteriocin production (De Vuyst & Leroy, 2007; Duarte-Villanueva et al., 2026), the inhibition of LAB to *Salmonella* in egg-based systems has not been systematically evaluated, and the minimum inhibitory concentrations required for pathogen inactivation remain unclear.

Additionally, strain-dependent metabolic activities may produce undesirable volatile compounds. For instance, some LAB generate excessive acetic acid, while other byproducts may contribute to fishy odors when exceeding sensory thresholds (Gao et al., 2025).

Strain-level safety also warrants attention. While many LAB species are generally recognized as safe, strain-dependent differences in metabolic activity and virulence potential exist (Zhang et al., 2026). Moreover, LAB may carry transferable antimicrobial resistance (AMR) genes that could be horizontally transmitted to pathogenic bacteria in the gut (Kaszab et al., 2023). Thus, strains intended for commercial egg fermentation should be carefully screened for AMR genes and safety status.

Regarding allergenicity, LAB fermentation has been reported to reduce the allergenicity of EW through proteolytic degradation (Pi et al., 2022). However, the extent of this reduction is strain- and condition-dependent, and residual or even novel allergenic potential may remain, warranting further investigation.

Finally, the stability of fermented egg products during storage requires attention. Post-fermentation acidification, water activity changes, and bacteriocin activity may influence the survival of spoilage organisms and pathogens, while LAB viability may decline, affecting both safety and probiotic benefits. Shelf-life criteria based on microbiological, chemical, and sensory parameters should be established.

In summary, despite the promising functional benefits of LAB-fermented egg products, their development is constrained by the inherent limitations of egg matrices as fermentation substrates, the scarcity of research on bioactive potential, the lack of understanding of bidirectional LAB–egg interactions, and multiple underexplored safety concerns. Future research should prioritize elucidating the mechanisms of bioactive compound production, unraveling the reciprocal regulation between egg components and LAB physiology, and systematically evaluating safety parameters including biogenic amines, pathogen control, strain-level safety, antimicrobial resistance, allergenicity, and storage stability to facilitate safe and effective commercialization.

## 9 Conclusion

LAB fermentation induces comprehensive transformations in egg matrices: proteins and lipids are partially hydrolyzed, while exogenous carbohydrates are metabolized into organic acids, flavor compounds, and free amino acids. These biochemical events drive structural alterations in egg proteins from macroscopic morphology to secondary and tertiary conformations, which in turn modulate PP including gel texture, rheology, emulsifying characteristics, foaming capacity, and surface hydrophobicity. Critically, the direction and magnitude of these changes are condition-dependent, governed by substrate composition, protein concentration, pH, and strain selection.

Compared with dairy matrices, egg systems impose greater constraints on LAB growth and metabolism due to carbohydrate deficiency, complex protein structures, and antimicrobial components such as lysozyme. Successful fermentation therefore requires optimization of protein concentration, dilution, carbon supplementation, and initial pH.

Current research remains limited in two respects: the bioactive potential of fermented egg matrices—particularly regarding potential gut-brain axis modulation—has not been adequately explored, and the reciprocal regulation of LAB physiology by egg-derived components remains largely unexplored. Future work should prioritize the formation mechanisms of bioactive compounds (e.g., GABA, bacteriocins, SCFAs) and the application of multi-omics to decipher bidirectional egg matrix–LAB interactions, thereby enabling the rational design of next-generation egg-based functional foods.

## CRediT authorship contribution statement

**Yang Zhou:** Writing – review & editing, Supervision. **Zhiwen Deng:** Writing – original draft. **Siyu Dai:** Writing – original draft. **Ruohan Yang:** Writing – original draft. **Yutong Xue:** Writing – original draft. **Huanhuan Wei:** Writing – original draft.

## Declaration of generative AI and AI-assisted technologies in the writing process

During the preparation of this work the authors used DeepSeek in order to improve the readability and language of the manuscript. After using this tool, the authors reviewed and edited the content as needed and take full responsibility for the content of the published article.

## Funding

This work was supported by 10.13039/501100004858Sichuan University of Science and Engineering under Grant 2024RC079.

## Declaration of competing interest

The authors declare that they have no known competing financial interests or personal relationships that could have appeared to influence the work reported in this paper.

## Data Availability

No data was used for the research described in the article.
